# Response to two Janus kinase inhibitors in a boy with SAVI during 2-year follow-up: case report and literature review

**DOI:** 10.3389/fimmu.2025.1615075

**Published:** 2025-07-08

**Authors:** Yiting Chen, Wenhe Zang, Haoyuan Zhong, Xianqin Deng, Wenting Zhong, Lianyu Wang, Xinying Chen

**Affiliations:** ^1^ Department of Pediatrics, The Second Affiliated Hospital of Guangzhou University of Chinese Medicine, Guangdong Provincial Hospital of Chinese Medicine, Guangzhou, China; ^2^ Youjia Xu’s Famous Expert Inheritance Studio of Guangdong Provincial Hospital of Chinese Medicine, Guangzhou, China; ^3^ Department of Radiology, The Second Affiliated Hospital of Guangzhou University of Chinese Medicine, Guangdong Provincial Hospital of Chinese Medicine, Guangzhou, China; ^4^ Department of Pediatrics, The Second Affiliated College of Guangzhou University of Chinese Medicine, Guangdong Provincial Hospital of Chinese Medicine, Guangzhou, China; ^5^ Mingzhao Du’s Inheritance Studio of Chinese Medicine of Guangdong Provincial Hospital of Chinese Medicine, Guangzhou, China; ^6^ Ziyuan Wen’s Academic Inheritance Studio of Chinese Medicine of Guangdong Provincial Hospital of Chinese Medicine, Guangzhou, China

**Keywords:** interstitial lung disease, pulmonary arterial hypertension, type I interferonopathy, STING1, Janus kinase inhibitors, ruxolitinib, tofacitinib, case report

## Abstract

STING-associated vasculopathy with onset in infancy (SAVI) represents an identified rare type I interferonopathy, triggered by gain-of-function mutations in the *STING1* gene. It is characterized by early-onset systemic inflammation, cutaneous vasculopathy, pulmonary involvement, and recurrent bacterial infections. When conventional treatments prove ineffective in managing clinical symptoms, a high index of suspicion and prompt genetic testing become pivotal in considering the potential therapeutic role of Janus kinase (JAK) inhibitors, with ruxolitinib and tofacitinib emerging as promising treatment options. Here, we present a case involving a patient with severe lung manifestations of SAVI, treated initially with tofacitinib and later switched to ruxolitinib due to inadequate response. During a 24-month follow-up period, while symptoms stabilized under ruxolitinib, chest computed tomography (CT) scans revealed progressive changes. This case report offers valuable insights into the use of JAK inhibitors in a patient with SAVI. It illustrates the complexities of managing such cases and underscores the need for continued investigation into novel therapeutic approaches.

## Introduction

STING-associated vasculopathy with onset in infancy (SAVI) represents an identified rare type I interferonopathy, triggered by gain-of-function mutations in the *STING1* gene (1). Characterized by early-onset systemic inflammation, it encompasses severe cutaneous vasculopathy, pulmonary involvement, and recurrent bacterial infections. Due to the rarity of the condition and the overlapping symptoms with connective tissue diseases (CTDs), SAVI is frequently misdiagnosed, leading to delays in appropriate treatment and contributing to increased morbidity and mortality (2). Standard treatments like disease-modifying antirheumatic drugs prove ineffective in managing SAVI. Instead, JAK inhibitors, notably baricitinib, emerge as promising alternatives, along with ruxolitinib or tofacitinib (1). In this report, we present a case of SAVI accompanied by severe pulmonary manifestations, managed with tofacitinib and subsequently ruxolitinib, with a 24-month follow-up to assess the treatment response.

## Case presentation

**Figure 1:**
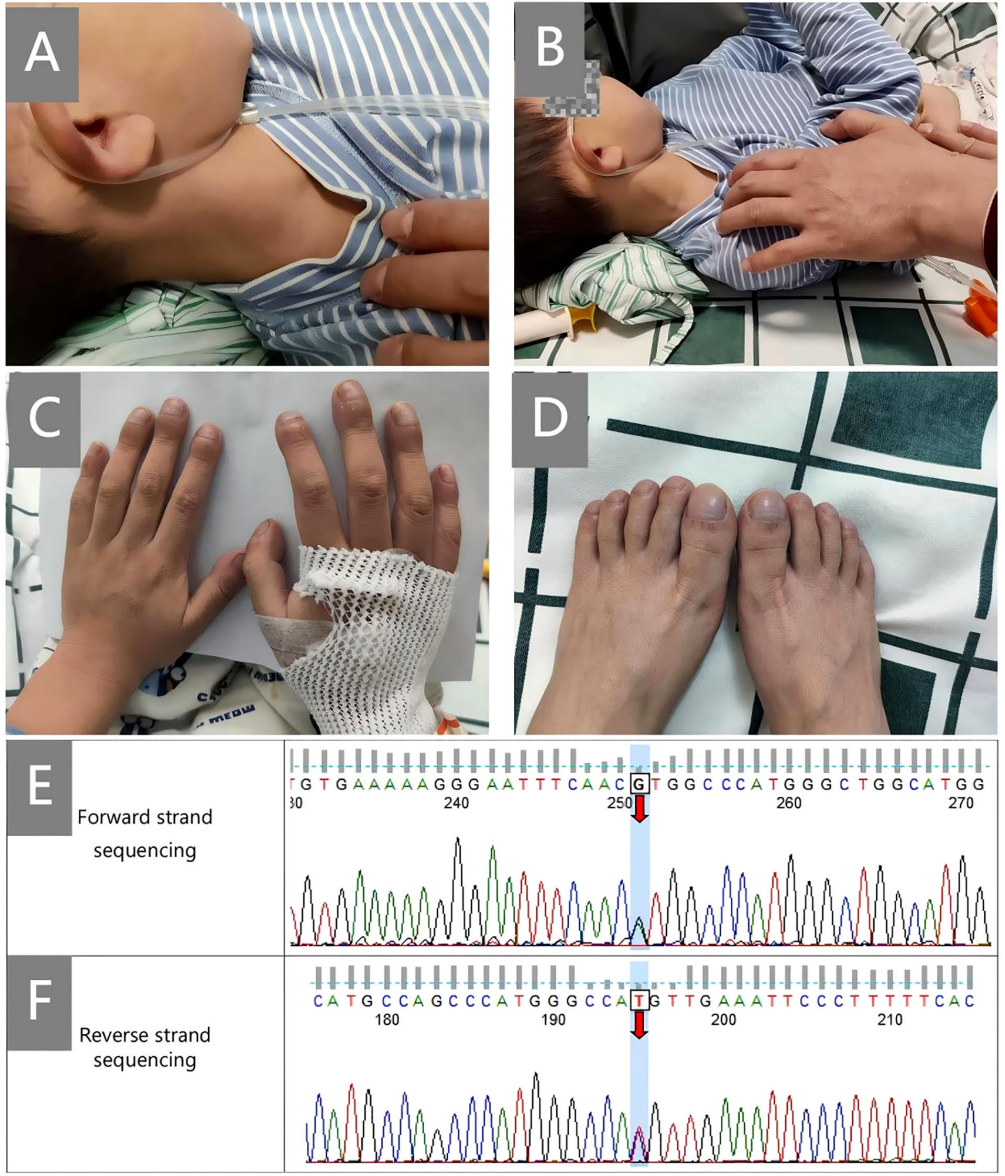
Clinical signs and Sanger sequencing map. **(A, B)**, hepatojugular reflux sign; **(C, D)**, clubbing; **(E, F)**, shows the confirmatory Sanger sequencing map of *STING1*. Forward and reverse strand sequencing were both used for the analysis. The numbers on the map are the numbers of the first-generation sequencing products, which are related to the designed primers and have nothing to do with the c. numbers in the gene naming.

A boy aged 5 years and 10 months, who had been experiencing recurrent cough and shortness of breath for 20 months, was admitted to our hospital due to symptom exacerbation. Physical examination revealed positive hepatojugular reflux sign and clubbing (**Figures 1A, B**, hepatojugular reflux sign; **Figures 1C, D**, clubbing). He is the third child of non-consanguineous parents, delivered via cesarean section at full term, weighing 3.6 kg at birth. His early development was normal regarding spirit, height, and weight, and there is no family history of autoimmune or rheumatic diseases.

The patient presented with alopecia areata at 2 years old and developed recurrent cough and fever at 4 years and 3 months. At the local hospital, he received a diagnosis of interstitial pneumonia, pulmonary hypertension, and right heart enlargement. Additional right heart catheter examination revealed pulmonary artery pressures of 60/15/31 mmHg, right atrium pressures of 8/0/3 mmHg, descending aorta pressures of 106/56/73 mmHg (systolic/diastolic/medium pressure), with a total pulmonary vascular resistance index of 45 dyne·s/cm^5^ (**Table 1**). Serological tests indicated positivity for multiple antibodies, including antinuclear antibody (ANA), β2GP1 immunoglobulin G (IgG), antiphospholipid immunoglobulin M (IgM), lupus anticoagulant, anti-cyclic citrullinated peptide antibody, anti-Ro-52 antibody, immunoglobulin A (IgA), and a positive Coombs’ test. Given the patient’s joint swelling and pain, further magnetic resonance imaging (MRI) for knee and rheumatoid factor (RF) tests were conducted, revealing left knee effusion with negative RF. Treatment with IVIG, prednisone (15 mg/day), mycophenolate mofetil, cyclophosphamide, pirfenidone, tadalafil, and selexipag led to improvement in cyanosis, and the alopecia areata and joint pain resolved completely without recurrence during follow-up. However, the patient’s condition progressed to dyspnea at rest (oxygen saturation, also called SpO2, 91-92%), necessitating home oxygen therapy.

**Table 1:** Important findings of the course.

Age	Important findings
4 years and 3 months	Right heart catheter examination (systolic/diastolic/medium pressure): pulmonary artery pressures of 60/15/31 mmHg, right atrium pressures of 8/0/3 mmHg, descending aorta pressures of 106/56/73 mmHg, with a total pulmonary vascular resistance index of 45 dyne·s/cm^5^.
	Serological antibodies: antinuclear antibody (ANA)/β2GP1 immunoglobulin G (IgG)/antiphospholipid immunoglobulin M (IgM)/lupus anticoagulant/anti-cyclic citrullinated peptide antibody/anti-Ro-52 antibody/immunoglobulin A (IgA): (+); Coombs' test: (+).
	Magnetic resonance imaging (MRI) for knee: left knee effusion; rheumatoid factor (RF): (-).
5 years and 10 months	Pulmonary function tests (before tofacitinib): VC max (Act/Pred): 33.87%; FVC (Act/Pred): 30.47%; FEV1 (Act/Pred): 31.46%; FEV1/FVC: 98.87%; impulse oscillometry (before tofacitinib): Z5 (Act/Pred): 96.7%; R5 (Act/Pred): 92.1%; R20 (Act/Pred): 67.4%; X5, 0.42 (Pred, -0.31[kPa/(L/S)]).
	Acute-phase reactants: CRP, 59.46 mg/L (reference: 0-6mg/L); ESR, 81 mm/h, reference (0-20mm/h); TNF-α, 15.10pg/mL (reference: 0-8.1pg/mL); PCT, 0.11 mg/L (reference: 0-0.06mg/L).
	Serological antibodies: IgA, 6.34 g/L (reference: 0.3-1.88g/L); IgG, 16.50 g/L (reference: 5.4-13.40g/L); ANA: (+), with a granular type titer of 1:320 and a cytoplasmic granular type titer of 1:100.
	T lymphocyte subset: CD4+/CD8+, 0.42 (reference: 0.55-2.09); percentage of CD4+T lymphocytes, 20.40% (reference: 28-47%); percentage of CD8+T lymphocytes, 48.22% (reference: 16-30%)
6 years and 10 months	Impulse oscillometry (12 months after tofacitinib): Z5 (Act/Pred): 163.6%; R5 (Act/Pred): 160.9%; R20 (Act/Pred): 121.6%; X5: -0.74 (Pred, -0.39[kPa/(L/S)]).

(+), positive; (-), negative; VC max, maximal vital capacity; FVC, forced vital capacity; FEV1, forced expiratory volume in one second; Z5, total respiratory impedance at 5 Hz; R5/R20, resistance at 5/20 Hz; X5, reactance at 5 Hz, Act/Pred, actual value/predicted value.

Pulmonary function tests uncovered severe restrictive ventilatory dysfunction, whereas arterial blood gas analysis indicated an oxygen partial pressure of 51.3 mmHg and a carbon dioxide partial pressure of 41.5 mmHg without oxygen inhalation, signifying type I respiratory failure. Laboratory investigations revealed leukocytosis, neutrophilia, and elevated levels of C-reactive protein (CRP), erythrocyte sedimentation rate (ESR), procalcitonin (PCT), and TNF-α. Regarding antibodies, both IgA and IgG were beyond the normal range, and ANA testing was positive, with a granular type titer of 1:320 and a cytoplasmic granular type titer of 1:100. Examination of the T lymphocyte subset uncovered a decreasing CD4+T to CD8+T lymphocyte ratio, accompanied by a reduced percentage of CD4+T lymphocytes (20.40%) and CD8+T lymphocytes (48.22%).

**Figure 2:**
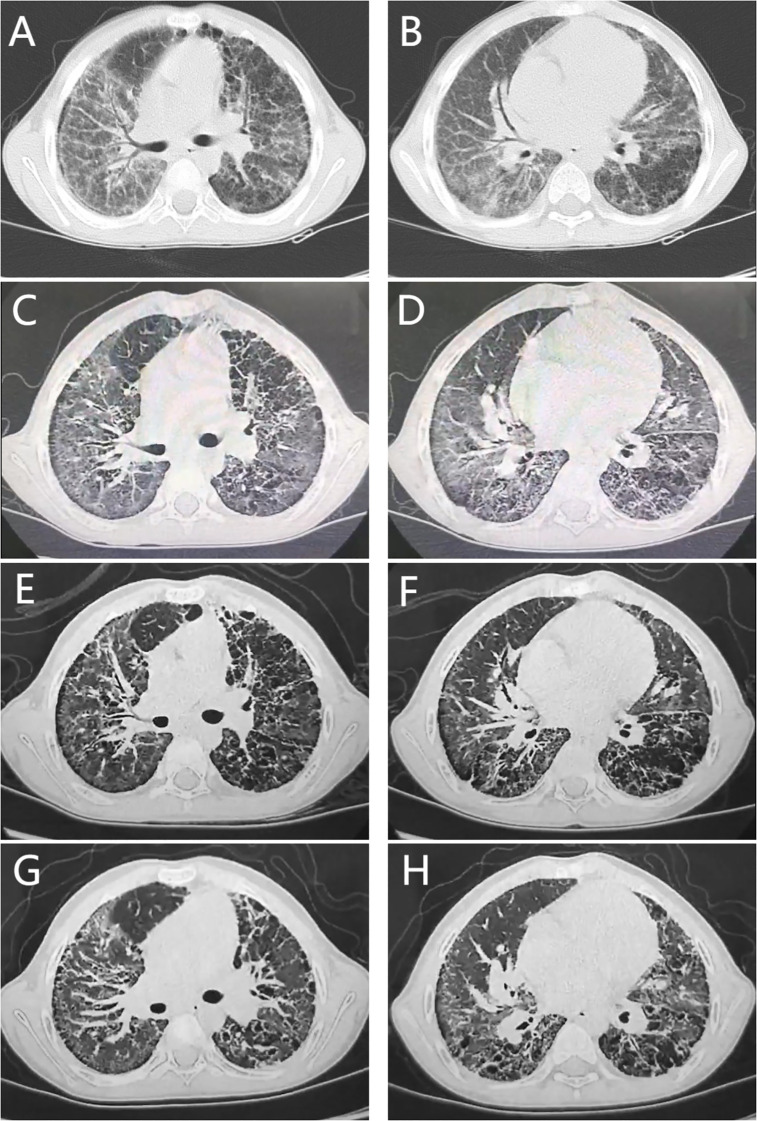
Chest computed tomography findings. **(A, B)**, the CT scan before tofacitinib. **(C, D)**, the CT scan 1 year after tofacitinib. **(E, F)**, the CT scan 2 months after ruxolitinib. **(G, H)**, the CT scan 1 year after ruxolitinib. All CT scans showed diffuse reticular shadows in the lungs, multiple subpleural microcystic lesions, and thickening of the interlobular septa. Neither tofacitinib nor ruxolitinib treatment could prevent the increase in the number and size of cystic shadows. **(E–H)** showed honeycombing changes and worsening emphysema.

Finally, through whole-exome sequencing (WES) and complementary forward and reverse Sanger sequencing analysis, a substitution was identified at the 463rd nucleotide (**Figures 1A, E–G**) of *STING1* in exon 5 (p.V155M; V: valine, M: methionine). Genetic testing of the parents subsequently confirmed this to be a *de novo* mutation. Analysis indicated that the missense variant was pathogenic (**Supplementary 1**), corroborating the diagnosis of SAVI. Initially, tofacitinib (3.3mg, twice a day) was administered for 12 months. However, there was no improvement in oxygen saturation, which remained at 91–92% on room air. The patient continued to experience dyspnea at rest, with a persistent Volpi’s dyspnea score of 4 (Volpi’s dyspnea score, ranked 0 to 4 (3)). High-resolution chest CT scans showed no amelioration of interstitial changes and, in fact, revealed progression of cystic lesions and honeycombing (**Figures 2A, B**, the CT scan before tofacitinib. **Figures 2C, D**, the CT scan 1 year after tofacitinib.). Additionally, after tofacitinib for 12 months, impulse oscillometry (IOS) indicated worsening pulmonary function, with increased airway resistance, central airway resistance, and peripheral elastic resistance compared to baseline values before initiating tofacitinib (**Table 1**). Therefore, the treatment was switched to ruxolitinib (5mg, twice a day), resulting in notable relief of resting dyspnea within one week, as reflected by an improvement in oxygen saturation (SpO2 at rest: 95-97%) and Volpi’s dyspnea score from 4 to 3 (dyspnea while walking). Continued clinical improvement over six months enabled the discontinuation of oxygen therapy, with the Volpi’s score further improving to 2 (dyspnea only during intense physical activity). Over the course of a year, Prednisone, mycophenolate mofetil, cyclophosphamide, pirfenidone, tadalafil, and selexipa were gradually reduced. However, during the follow-up, there was no progress in lung function. Neither tofacitinib nor ruxolitinib proved effective in halting the growth in number and size of cystic shadows. CT images showed honeycombing alterations and advancing emphysema (**Figures 2E, F**, the CT scan 2 months after ruxolitinib; **Figures 2G, H**, the CT scan 1 year after ruxolitinib.). Unfortunately, due to the inconvenience, IOS or other pulmonary function tests were not conducted following ruxolitinib initiation. Currently, the patient remains in stable condition, experiencing breathing difficulties only during intense physical activity.

## Discussion

SAVI, a rare type I interferonopathy first reported in 2014, arises from gain-of-function mutations in the *STING1* gene, mainly inherited in an autosomal dominant manner, with a few cases of autosomal recessive inheritance and somatic mosaic mutations (1, 2, 4, 5). This condition is characterized by early-onset systemic inflammation, pronounced cutaneous vasculopathy, pulmonary involvement, and recurrent bacterial infections. SAVI is often misdiagnosed as a connective tissue disease (CTD), which can delay appropriate treatment and contribute to increased morbidity and mortality, as it typically responds poorly to conventional antirheumatic therapies (2). In this report, we present a case involving a child with SAVI, caused by the *de novo* c.463 G>A variant. The patient exhibited early-onset interstitial lung disease (ILD) and pulmonary arterial hypertension (PAH) as key clinical signs. Despite a year of tofacitinib treatment, the child’s cough, wheezing, and hypoxia persisted. Switching to ruxolitinib led to significant dyspnea improvement within a week, enabling the discontinuation of oxygen therapy after six months. However, chest CT scans revealed an increase in cystic lesions, honeycombing changes, and emphysema progression at 2 months and 1 year following ruxolitinib initiation.

Lung involvement stands out as a significant characteristic in SAVI patients, yet its initial symptoms are subtle, often presenting as early-onset progressive dyspnea, tachypnea, and/or cough. Notably, about one-third of patients require oxygen therapy either at the onset or during the disease progression, and some may even advance to end-stage respiratory failure in adolescence, necessitating lung transplantation (2, 6). Pulmonary function tests typically reveal restrictive or mixed ventilatory dysfunction, while obstructive ventilatory dysfunction is observed in approximately 10% of patients (2, 7–9). Chest CT scans commonly exhibit random asymmetric ground-glass opacities and cystic lesions accompanied by diffuse peripheral lobular septal thickening, distinguishing them from ILD linked to connective tissue disease (2, 9, 10). An escalation in both the number and size of cystic lesions signifies the advancement of fibrosis (2, 11). Our patient, who suffered from recurrent cough and dyspnea, required home oxygen therapy. Pulmonary function tests and chest CT findings aligned with typical SAVI manifestations. However, the patient developed pulmonary hypertension, a rarity in SAVI with a reported incidence of less than 10%, possibly attributed to severe disease progression (2, 11).

SAVI is a multisystem disease where skin involvement is a frequent presenting symptom. Common skin manifestations include chilblain-like rash, erythema, purpura, desquamative rash, and gangrene, among others. Additionally, alopecia totalis can emerge as an atypical marker of skin damage (2, 6, 12). Our patient exhibited mild skin symptoms, specifically alopecia areata, which appeared at 2 years of age. Reports indicate that patients with minor skin involvement may still develop severe lung disease, aligning with our patient’s presentation (8, 13). Approximately one-third of patients encounter joint problems, often polyarthritis linked to RF positivity, and potentially leading to destructive joint damage (2, 14). Our patient experienced joint swelling, pain, and effusion; however, RF was negative, and symptoms resolved following traditional immunosuppressive therapy. Children with chronic illnesses, including SAVI, frequently encounter growth retardation. Moreover, high-dose glucocorticoids, besides the disease’s natural effects, can contribute to obesity and metabolic disturbances. SAVI may also be accompanied by rare manifestations such as necrotizing hepatitis, cholangitis, cardiac hypertrophy, epicardial ischemia, pericarditis, and proteinuria (2, 6, 15, 16).

SAVI is a systemic inflammatory disease distinguished by specific laboratory markers, including elevated CRP and ESR levels, hyperimmunoglobulinemia marked by increased IgG and IgA levels, and the existence of various positive autoantibodies, such as ANA, ANCA, antiphospholipid antibodies, lupus anticoagulants, and anti-double-stranded DNA antibodies. Furthermore, there’s a decrease in CD3+ and CD4+ T cell lymphocytes accompanied by an elevation in CD8+ T cell lymphocytes. These overlapping characteristics often lead to the misdiagnosis of SAVI as lupus, ANCA vasculitis, or undifferentiated connective tissue disease (2, 6, 9, 13). When conventional treatments prove ineffective in managing clinical symptoms, early suspicion and genetic testing become crucial to explore the potential use of JAK inhibitors in therapy. Notably, ruxolitinib and tofacitinib stand out as viable therapeutic alternatives (11).

Based on the literature review presented in **Table 2**, among the 11 patients treated with tofacitinib (6, 7, 14, 17–21), 8 exhibited lung involvement (6, 7, 17–20). While 6 patients showed improvement in respiratory symptoms (17–20), 2 patients did not experience any improvement in lung damage after treatment (3, 4). Notably, 2 patients who initially showed respiratory improvement later had worsened CT scans, and one patient, unfortunately, succumbed to acute respiratory failure at the age of 20 months during an influenza outbreak (reference 16). All patients presented with skin symptoms (6, 7, 14, 17–21), with 8 patients demonstrating rash improvement post-treatment (7, 14, 19–21). However, one patient’s rash worsened 3 months after treatment, necessitating additional prednisone therapy (19), while rash changes in 2 patients remained unclear after treatment (6, 18). Among the 12 patients treated with ruxolitinib (3, 8, 20, 22–24), 5 responded poorly. One patient died due to ILD and heart failure (8), another showed no improvement in lung and joint symptoms (8), and three patients required lung transplantation (23). A notable case was a patient with skin and lung involvement and global developmental delay who showed significant improvement in respiratory symptoms, mobility, gross and fine motor skills, and language after receiving ruxolitinib combined with prednisolone and anti-pulmonary hypertension treatment. Although his weight increased from below the 3rd percentile to the 20th percentile 5 months after treatment, a cyanotic rash persisted, and he lost his nasal septum completely 6 months post-treatment (22). In 6 other cases, both skin and lung symptoms improved (3, 20, 24) and in 2 patients, steroids were reduced or discontinued after treatment (3, 20). However, the efficacy was not sustained in some patients. One patient’s ILD worsened 18 months after treatment (3), and another patient experienced a relapse of lung disease 10 months post-treatment, requiring an additional 2 mg/kg/day of prednisone (3). Both tofacitinib and ruxolitinib demonstrated some degree of improvement in skin and lung symptoms, but the relief of SAVI symptoms was not consistently satisfactory.

**Table 2:** Characteristics of literature review.

Author(year/country)	Age of onset/gender/Age of diagnosis	HGVSc/ HGVSp	Skin lessions	Pulmonary involvement	Other Manifestations	JAK Inhibitors	Response to JAK Inhibitors and Prognosis
Shen D (2022/ China) (17)	8m/M/19m	c.463G>A/ p.V155M (*de novo*)	facial rash; chilblain-like rash on the auricles; recurrent oral ulcers	ILD	developmental delay (height <-3SD; weight <-2SD, >-3SD)	tofacitinib(2.5mg, bid)	Following a three-month course of tofacitinib therapy, the skin lesions resolved, and a high-resolution chest CT scan revealed an improvement in ILD. Despite these positive outcomes, recurrent oral ulcers and growth retardation remained unchanged, and there was no notable progress in serum immunoglobulins or immune phenotype.
Yu ZX (2018/ China) (18)	After birth/M/14y	c.463G>A/ p.V155M (*de novo*)	recurrent chilblain-like rash; telangiectasia; hair loss	ILD; PAH; hypoxemia; restrictive ventilatory dysfunction	right heart enlargement; developmental delay (height at P3-P10; weight < P3)	tofacitinib (5mg, bid)	Two weeks later, he no longer had hypoxemia, his activity tolerance increased, and he could sustain moderate-intensity exercise for more than 15 minutes.
Tang X (2020/ China) (19)	3m/F/12m	c.463G>A p.V155M (*de novo*)	chilblain-like rash; telangiectasia	ILD	recurrent fever; myositis; developmental delay	tofacitinib(2.5mg,bid)	In the early stage, rash and respiratory symptoms showed improvement, yet recurrent fevers persisted. CT scans indicated progression of the disease, and ultimately, the patient succumbed to acute respiratory failure at the age of 20 months during an outbreak of influenza.
	54m/M/60m	c.463G>A/ p.V155M (*de novo*)	telangiectasia; erythema; purpura; scaly rash	ILD	recurrent fever; arthritis; ureteral stones; cerebrovascular involvement	tofacitinib(2.5mg,bid)	During the initial four months of treatment, the patient experienced improvement in respiratory symptoms; however, CT findings deteriorated, while joint pain resolved. Following three months of therapy, the rash intensified, necessitating treatment with prednisone at 1mg/kg for its amelioration. Ten months later, tofacitinib was discontinued spontaneously, and the prednisone dosage was decreased. Notably, respiratory and joint symptoms remained stable, yet the rash worsened, and the patient continued to survive.
Tokgun PE (2023/ Turkey) (21)	U/F/45y (mother)	c.580G>T/ p.V194L	multiple skin swelling; bruises	---	rheumatoid arthritis; psoriatic arthropathy	tofacitinib(5mg,bid)	All symptoms were reduced by approximately 70% within two weeks and completely disappeared after one month of treatment, and tofacitinib treatment has been continued for more than a year.
	U/F/20y (daughter)	c.580G>T/ p.V194L (inherited from mother)	multiple skin swelling; bruises	---	rheumatoid arthritis; psoriatic arthropathy	tofacitinib(5mg,bid)	Within two weeks, all symptoms had decreased by roughly 70%, and they vanished entirely after a month of treatment. The patient has been continuously treated with tofacitinib for over a year now.
Kim HR (2024/ Korea) (6)	2m/M/29y	c.439G>A/ p.V147M (*de novo*)	multiple erythema and pustules; chilblain-like rash, pustular psoriasis; nasal septal perforation; foot gangrene	ILD; hypoxemia; restrictive ventilatory dysfunction	proteinuria, class II lupus nephritis of kidney biopsy	tofacitinib(dosage not mentioned)	The patient’s lung condition did not improve after treatment with tofacitinib. The patient’s mobility was severely limited due to breathing difficulties and he eventually required a lung transplant.
Seo J (2017/ Korea) (7)	6m/M/9y	p.S102P +p.F279L (inherited from father)	telangiectasia with gangrenous lesions; nasal septal perforation	bronchiolitis obliterans with peribronchial inflammation; hypoxemia	acute cerebral infarction and subarachnoid hemorrhages	tofacitinib(5mg/day)	After three months of tofacitinib treatment, the patient's telangiectatic skin lesions showed improvement, while the lung damage remained unchanged.
Barry KK (2024/ America) (14)	U/M/5y	p.R284T (*de novo*)	reddish-purple reticular plaques on the face and extremities; edema of the hands and feet; scarring of the toes; saddle nose deformity; sparse hair	---	developmental delay (height and weight <P1), severe congenital neutropenia; perirectal abscesses; inflammatory polyarthritis	tofacitinib (3.2mg,bid)	After six months of tofacitinib treatment, the patient showed clinical improvement in skin symptoms, with ulcers healing, hair regenerating, and weight gain observed. However, progressive destructive arthritis persisted, leading to a switch back to adalimumab, methotrexate, azathioprine, and hydroxychloroquine for further management.
Volpi S (2019/ Italy) (3)	8m/F/9y	c.463G>A/ p.V155M (*de novo*)	erythematosus-infiltrated skin lesions with pustular evolution; scarring; chilblains; nail dystrophy	ILD; mixed ventilatory dysfunction	recurrent fever, microscopic hematuria, mild proteinuria	ruxolitinib(0.65mg/kg/day)	Clinical symptoms showed improvement within a few weeks and markedly improved after three months. This progress was evident in the amelioration of lung disease, complete resolution of skin lesions, disappearance of microscopic hematuria, and the eventual discontinuation of steroids after two years.
	3m/F/7y	c.842G>A/ p.R281Q	malar rash, livedo reticularis on lower limbs	ILD; PAH; hypoxemia	recurrent lower respiratory tract infections; developmental delay; hypertension	ruxolitinib(0.7mg/kg/day)	In the early stage of ruxolitinib treatment, skin symptoms improved, dyspnea was relieved, blood oxygen saturation increased (84% to 98%), echocardiography showed improvement in PAH, and pulmonary artery size returned to normal; hospitalization due to recurrent respiratory tract infection after 7 months of treatment; ILD lesions worsened after 18 months of treatment.
	3d/F/3y	c.461A>G/ p.N154S (*de novo*)	multiple erythematosus vesicular rash, pustules and scars	ILD	---	ruxolitinib(1.25mg/kg/day)	In the early stage of ruxolitinib treatment, skin lesions and lung imaging improved; lung disease recurred after 10 months of treatment, requiring the addition of prednisone (2 mg/kg/day).
Li W (2022/ China) (20)	3m/M/1y3m	p.N154S	chilblain-like rash; ulcers; erythema	ILD; hypoxemia	recurrent fever; developmental delay (weight < -2SD)	tofacitinib (0.5mg/kg/day), ruxolitinib (0.5mg/kg/day)	After tofacitinib treatment, the fever subsided, the rash improved, which only appeared in winter and was mild in severity and duration, with little restriction in daily activities, and chest CT improved. Due to unsatisfactory control of CRP and ESR, tofacitinib was replaced with ruxolitinib after 15 months of treatment, and the median dose of prednisone was reduced to 0.20 (0.11-0.36) mg/kg/day, and the autoantibody level decreased.
	6m/M/12y10m	p.V155M	chilblain-like rash; telangiectasia	ILD; PAH; hypoxemia; severe restrictive lung dysfunction	right ventricular enlargement; developmental delay (weight < -2SD)	tofacitinib (0.38mg/kg/day)	The rash completely disappeared and steroids were discontinued. Daily activities were basically unrestricted, PAH improved, and anti-PAH treatment was still maintained. There was no significant change in chest CT, but the level of autoantibodies decreased.
Saldanha RG (2018/ Australia) (22)	2m/M/3y	c.850A>G/ p.R284G (*de novo*)	erythematous papules, blisters, and livedo reticularis on the limbs	ILD; PAH	global developmental delay	ruxolitinib(5mg/day)	After 8 weeks of ruxolitinib combined oral prednisolone (2 mg/kg/day) and anti-pulmonary hypertension treatment, respiratory symptoms and mobility improved, and gross motor and fine motor skills and language improved rapidly; 5 months after treatment, body weight increased from <P3 to P20; however, the lividinous rash persisted, and the nasal septum was completely lost 6 months after ruxolitinib treatment.
Wang Y (2021/ China) (8)	36y8m/M/37y (father)	c.841G>A/ p.R281Q (*de novo*)	nail dystrophy	ILD; PAH; hypoxemia;severe restrictive lung dysfunction	---	ruxolitinib(0.2mg/kg/day)	The escalation of the ruxolitinib dose was poorly tolerated, and the patient died 4 months later due to ILD progression and heart failure.
	2y/M/13y (son)	c.841G>A/ p.R281Q(AD)	---	ILD; restrictive lung dysfunction	polyarthritis, joint pain, developmental delay (height and weight < P3)	ruxolitinib(0.16mg/kg/day)	The escalation of ruxolitinib dose was poorly tolerated, with no improvement in arthritis, arthralgia, or chest CT.
Berrada KR (2023/ France) (23)	5y/M/12y	p.V155M (AD)	chilblain-like rash; telangiectasia	ILD; hypoxemia;	recurrent fever	ruxolitinib(0.4mg/kg/day)	Despite treatment with ruxolitinib, his respiratory status continued to deteriorate until he had recurrent pneumothoraces, requiring bilateral lung transplant (BLT) at age 17. He was still alive 2.6 years after BLT.
	1y/F/12y	p.V155M (*de novo*)	---	ILD; hypoxemia;	arthritis; developmental delay	ruxolitinib(0.3mg/kgday)	In spite of treatment with ruxolitinib, the patient's respiratory status progressed, with worsening ILD on CT and hypoxemia, requiring lung transplantation at age 13.9 years, and died 4 years after BLT due to left bronchial rupture.
	0.7y/M/14y	p.V155M (*de novo*)	chilblain-like rash; telangiectasia	ILD;PAH; hypoxemia	---	ruxolitinib(0.3mg/kgday)	Despite treatment with ruxolitinib, the patient's respiratory status progressively deteriorated, requiring lung transplant, and he died 33 days after the second transplant at the age of 16 due to multiorgan failure and hemorrhagic shock.
Alghamdi MA (2021/ Saudi Arabia) (24)	U/M/15y (brother)	c.841C>T/ p.R281W (AR)	nasal rash and nasal deviation; acrocyanosis; nail dystrophy; dorsal hyperpigmentation; alopecia areata	ILD;PAH; hypoxemia	Severe right ventricular dilatation with moderate right ventricular dysfunction	ruxolitinib (5mg, bid)	The patient's pulmonary and skin symptoms improved significantly after ruxolitinib treatment. After 6 months, he no longer had dyspnea at rest. He did not require oxygen therapy with decreased immunoglobulin levels and acute phase reactants, a reduction in the size of the area of hair loss with hair regrowth, and a decrease in the hypopigmentation.
	U/F/7y (sister)	c.841C>T/ p.R281W (AR)	malar rash with Raynaud’s phenomenon	ILD; PAH	arthritis	ruxolitinib(5mg, bid)	The patient's pulmonary and skin symptoms improved significantly after ruxolitinib treatment, and after 6 months, she no longer had dyspnea at rest.

SD, standard deviation; P, percentage; U, unmentioned; bid, bisindie; m, month; y, year; ---: no other involvement.

HGVSc, human genome variation society coding; HGVSp, human genome variation society protein; AD, autosomal dominant inheritance; AR, autosomal recessive inheritance.

The abbreviations of HGVSc/ HGVSp variants: V, valine; M, methionine; S, serine; P, proline; F, phenylalanine; L, leucine; T, threonine; N, aspartic acid; S, serine; R, arginine; Q, glutamine; W, tryptophan; ILD, interstitial lung disease; PAH, pulmonary arterial hypertension.

Notably, in **Table 2**, most patients carried the p.V155M variant in the *STING1* gene, while others had variants such as p.V194L, p.V147M, p.S102P + p.F279L, p.R284T, or p.R281Q. We further analyzed the clinical features and treatment outcomes of patients harboring the p.V155M variant. All patients with this variant exhibited lung involvement (3, 17–20, 23). Skin manifestations were present in 8 patients (80%) (3, 17–20, 23), developmental delay in 6 (60%) (17–19, 23), and recurrent fever in 5 (50%) (3, 19, 23). Early onset (before 1 year of age) occurred in 8 patients (80%), while only 2 patients (20%) had disease onset after the age of 4 years. However, only 3 patients were diagnosed before the age of 4, all of whom demonstrated improved respiratory symptoms following treatment with tofacitinib (17, 19). Unfortunately, one of these patients experienced radiologic worsening and died from acute respiratory failure at 20 months of age during an influenza outbreak (19). Among the 7 patients diagnosed after the age of 4 (3, 18–20), 3 received tofacitinib and showed clinical improvement in respiratory symptoms (18–20), though one also exhibited radiologic progression (19). The remaining 4 patients were treated with ruxolitinib (3, 23), and only one showed substantial clinical improvement within a few weeks, which continued over 3 months and included resolution of lung disease, cutaneous lesions, and microscopic hematuria, allowing steroid discontinuation after 2 years (3). However, 3 patients (3/4, 75%) eventually required lung transplantation (23). Of these, one survived 2.6 years post-transplant, one died 4 years later, and one died 33 days after a second transplant. These findings suggest that the effectiveness of JAK inhibitors in improving respiratory symptoms is greater when patients are diagnosed before the age of 4 (23). Our patient, who developed respiratory symptoms at 4 years and 3 months and was diagnosed at 5 years and 10 months, had a suboptimal response to tofacitinib. Following a switch to ruxolitinib after 12 months of ineffective therapy, the patient’s clinical symptoms stabilized, but chest CT continued to show progressive deterioration. Therefore, the variability in treatment effects may be attributed to factors such as race, gene mutation site, clinical phenotype, disease severity, age of onset, and age of diagnosis.

We conducted a deeper investigation into the reactions of Chinese SAVI patients to two JAK inhibitors. A Chinese boy, newly diagnosed with a c.463G>A mutation, exhibited recurrent facial rash since infancy, recurrent oral ulcers, chronic cough, and developmental delays. He commenced tofacitinib therapy (2.5 mg, twice daily) at 19 months. After three months, while recurrent oral ulcers and growth retardation persisted, his rash symptoms eased, and high-resolution chest CT indicated improvements in ILD (17). Another case involved a 14-year-old Chinese boy who displayed symptoms from birth, primarily recurrent dry cough and reduced activity tolerance, along with growth restriction, recurrent chilblain-like rash, telangiectasia, and clubbing. High-resolution chest CT revealed ILD, and echocardiography showed pulmonary hypertension and right heart enlargement. Sanger sequencing detected the *de novo* c.463G>A mutation. Following tofacitinib treatment (5 mg, twice daily), the patient’s activity tolerance enhanced (18). However, in two children with newly identified heterozygous mutations (c.463G>A, p. V155M), tofacitinib provided limited benefit for their ILD, with CT scans indicating deterioration in both cases with poor rash treatment response in one and fatality in another (19). A SAVI patient (p.N154S) presenting with skin and lung involvement, hypoxemia, and recurrent fever, showed fever resolution and rash improvement post-treatment. The rash was winter-specific, less severe, and short-lived. Daily activities were minimally restricted, and chest CT showed improvements. However, due to inadequate control of CRP and ESR, tofacitinib was substituted with ruxolitinib after 15 months. The median prednisone dose was reduced to 0.20 (0.11-0.36) mg/kg/day, leading to decreased autoantibody levels (20). Another SAVI patient (p.V155M) with pulmonary arterial hypertension (PAH) responded favorably to tofacitinib, with skin symptoms resolving, respiratory symptoms significantly improving, and daily activities becoming unrestricted. Chest CT remained unchanged, steroids were discontinued, PAH improved (though anti-PAH treatment continued), and autoantibody levels decreased (20). Two familial SAVI patients exhibited poor responses to ruxolitinib. The father, with a *de novo* c.841G>A mutation, succumbed to progressive ILD and heart failure, while the son, who inherited the mutation, showed no improvement in arthritis, joint pain, or chest CT findings (8). Drawing from our experience with Chinese patients, our subject sequentially received tofacitinib and ruxolitinib. Though tofacitinib was ineffective, ruxolitinib significantly alleviated clinical symptoms.

The *STING1* gene encodes the stimulator of interferon genes (STING), a key component of the cGAS-STING pathway alongside cyclic GMP-AMP synthase (cGAS). This pathway is essential for sensing exogenous DNA and mounting an innate immune response. Normally, pathogenic microorganisms or cell damage trigger the abnormal accumulation of double-stranded DNA (dsDNA) in the cytoplasm. cGAS then binds to dsDNA, catalyzing the production of 2′3′ cyclic GMP-AMP (cGAMP), a second messenger. The binding of cGAMP to STING initiates a conformational change, oligomerization, and activation of STING (5, 25). Activated STING recruits TANK-binding kinase 1 (TBK1) and activates interferon regulatory factor 3 (IRF3), leading to its dimerization and translocation to the nucleus. This, in turn, induces the expression of type I interferons, such as IFNα/β, and other inflammatory mediators. When IFN-I binds to its receptor, it activates JAK, upregulating the transcription of interferon-stimulated genes. Mutations in *STING1* can lead to the expression of IFN-I, bypassing the need for cGAMP activation, which characterizes the SAVI pathway (25). Additionally, activated STING promotes M1 macrophage polarization and regulates T cell proliferation and differentiation via non-IFN-I pathways, resulting in a significant increase in Th1 cells producing IFNγ—another crucial factor in SAVI pathogenesis (26, 27). IFNα/β signals through JAK1/tyrosine kinase 2 (Tyk2), whereas IFNγ activates JAK1/JAK2. Ruxolitinib is an inhibitor of JAK1 and JAK2, respectively, while tofacitinib primarily inhibits JAK1 and JAK3 and, to a lesser extent, JAK2 and Tyk2. Given their specificity, ruxolitinib is more suitable for SAVI treatment than tofacitinib. STING also mediates transcriptional activation via the nuclear transcription factor (NF-κB), although the mechanism of this pathway remains elusive (25). These pathways are implicated in cell apoptosis, pyroptosis, and senescence, and play a pivotal role in immune inflammation and tissue damage (10, 25–27). JAK inhibitors fail to suppress non-IFN-dependent inflammatory pathways, explaining why SAVI patients treated with these agents do not achieve complete remission. Moreover, pre-existing organ damage remains unrepaired. Hence, controlling inflammation, delaying end-organ damage, and preserving function are paramount. Currently, there is no consensus on the use of ruxolitinib in SAVI treatment. Our case study offers insight into replacing ruxolitinib in patients who respond poorly to tofacitinib. Although ruxolitinib improves clinical symptoms, it does not significantly alter chest CT findings. Future research should explore alternative SAVI treatment strategies, such as monoclonal antibody inhibitors targeting *STING1*, to enhance disease prognosis (10).

## Conclusions

We present a case involving a patient with severe lung manifestations of SAVI, treated initially with tofacitinib and later switched to ruxolitinib due to inadequate response. Over a 24-month follow-up period, while symptoms stabilized under ruxolitinib, chest CT scans revealed progressive changes. The complexities of diagnosing and managing SAVI over the long term pose significant challenges for clinicians. Notably, JAK inhibitors, while partially effective, have not fully arrested the inflammatory process or reversed organ damage in this case, highlighting the need for continued investigation and refinement of therapeutic approaches. Future research efforts are imperative.

## Data Availability

The original contributions presented in the study are included in the article/**Supplementary Material**. Further inquiries can be directed to the corresponding author.
