# Supplementary material for: Response to two Janus kinase inhibitors in a boy with SAVI during 2-year follow-up: case report and literature review
Source: Front Immunol. 2025 Jul 8;16:1615075. doi: 10.3389/fimmu.2025.1615075 (PMC12280360; doi:10.3389/fimmu.2025.1615075)
Supplement: Supplementary file 1 [file Table1.docx]

**Supplement 1. Results of the potential pathogenicity of missense variant.**

| Tools/ Criteria | Prediction | Score/Frequency* |
| --- | --- | --- |
| CADD | Uncertain | 25 |
| MutPred | Uncertain | 0.47 |
| PolyPhen | Possibly Damaging | 1.0 |
| SIFT | Pathogenic | 0.0 |
| M-CAP | Uncertain | 0.24 |
| Mutation assessor | Uncertain(Medium) | 2.83 |
| MutationTaster | Deleterious | 1 |
| FATHMM_MKL | Uncertain | 0.80 |
| PROVEAN | Benign | -1.6 |
| REVEL | Pathogenic | 0.74 |
| AlphaMissense | Uncertain | 0.57 |
| ClinPred | Pathogenic | 0.97 |
| GERP RS | Pathogenic | 3.7 |
| ClinVar | Pathogenic | --- |
| ACMG/AMP criteria | Pathogenic (PS4+PM1+PM2+PM6+PP3) | --- |

The results of online analysis tools showed that 50% (7/14) supported the pathogenicity of the variant c.463G>A, among which PolyPhen, SIFT, MutationTaster, REVEL, ClinPred, GERP RS, and ClinVar supported Pathogenic. CADD, MutPred, M-CAP, AlphaMissense, Mutation assessor, and FATHMM_MKL believed that its pathogenicity was unclear, and only PROVEAN predicted that the mutation was benign. Referring to the joint standard for genetic variation classification in the ACMG guidelines, the variant was pathogenic.
